# Assessment of Commercial and Mandatory Discounts in the Gross-to-Net Bubble for the Top Insulin Products From 2012 to 2019

**DOI:** 10.1001/jamanetworkopen.2023.18145

**Published:** 2023-06-14

**Authors:** Sean R. Dickson, Nico Gabriel, Walid F. Gellad, Inmaculada Hernandez

**Affiliations:** 1 West Health Policy Center, Washington, DC; 2 Division of Clinical Pharmacy, Skaggs School of Pharmacy and Pharmaceutical Sciences, University of California, San Diego, La Jolla; 3 Division of General Internal Medicine, University of Pittsburgh School of Medicine, Pittsburgh, Pennsylvania; 4 Veterans Affairs Pittsburgh Healthcare System, Pittsburgh, Pennsylvania

## Abstract

**Importance:**

Insulin list prices have grown substantially since 2010, but net prices have declined since 2015 because of manufacturer discounts, leading to an increasingly large difference between list and net prices of drugs often called the *gross-to-net bubble*. It remains unclear to what extent the gross-to-net bubble represents voluntary manufacturer discounts negotiated in commercial and Medicare Part D markets (hereafter called *commercial discounts*) vs mandatory discounts under the Medicare Part D coverage gap, Medicaid, and the 340B program.

**Objective:**

To decompose the overall gross-to-net bubble of leading insulin products into discount types.

**Design, Setting, and Participants:**

This economic evaluation obtained data from Medicare and Medicaid claims and spending dashboards, Medicare Part D Prescriber Public Use File, and SSR Health for the top 4 commonly used insulin products: Lantus, Levemir, Humalog, and Novolog. The gross-to-net bubble, which represents total discounts, was estimated for each insulin product and year (from 2012 to 2019). Analyses were conducted in June to December 2022.

**Main Outcomes and Measures:**

The gross-to-net bubble was decomposed into 4 discount types: (1) Medicare Part D coverage gap discounts, (2) Medicaid discounts, (3) 340B discounts, and (4) commercial discounts. Coverage gap discounts were estimated using Medicare Part D claims data. Medicaid and 340B discounts were estimated using a novel algorithm that accounted for best prices set by commercial discounts.

**Results:**

Total discounts for the 4 insulin products increased from $4.9 billion to $22.0 billion. Commercial discounts represented a majority of all discounts, increasing from 71.7% of the gross-to-net bubble in 2012 ($3.5 billion) to 74.3% ($16.4 billion) in 2019. Among mandatory discounts, coverage gap discounts remained relatively consistent as a proportion of discounts (5.4% in 2012 vs 5.3% in 2019). Medicaid rebates decreased as a proportion of total discounts, from 19.7% in 2012 to 10.6% in 2019. The 340B discounts increased as a proportion of total discounts from 3.3% in 2012 to 9.8% in 2019. Results for the contribution of discount types to the gross-to-net bubble were consistent across insulin products.

**Conclusions and Relevance:**

Results of a decomposition of the gross-to-net bubble for leading insulin products suggest that commercial discounts play a growing role in lowering net sales compared with mandatory discounts.

## Key Points

**Question:**

What proportion of discounts for insulin products is accounted for by mandatory discounts vs voluntary commercial discounts that are negotiated between manufacturers and pharmaceutical benefit managers?

**Findings:**

In this economic evaluation of the 4 leading insulin products, discounts increased from $4.9 billion to $22.0 billion or 37.6% to 81.4% of gross sales from 2012 to 2019. Voluntary, negotiated discounts represented most of the discounts and increased from 27.0% to 60.5% of gross sales over the study period. Mandatory discounts under Medicare Part D coverage cap, Medicaid, and 340B programs represented the remaining discounts.

**Meaning:**

Findings of this study suggest that commercial discounts for insulin products play a growing role in lowering net sales compared with mandatory discounts.

## Teaser

This economic evaluation examines the proportion of discounts for insulin products accounted for by mandatory discounts vs voluntary commercial discounts that are negotiated between manufacturers and pharmaceutical benefit managers.

## Introduction

Insulin list prices have increased substantially faster than overall brand drug prices since 2010,^1^ but net prices have declined since 2015 because of rising manufacturer discounts.^2,3^ These diverging patterns have played a role in the increasingly large differences between list and net prices, called the *gross-to-net bubble*.^1^ The gross-to-net bubble comprises voluntary discounts (hereafter called *commercial discounts*), which are negotiated between manufacturers and pharmacy benefit managers in the commercial and Medicare Part D markets, as well as mandatory discounts under the Medicare Part D coverage gap, Medicaid, and 340B programs.

Previous studies documented the magnitude of the gross-to-net bubble but did not decompose the overall difference into components due to the difficulty of estimating the magnitude of each discount.^1^ Understanding patterns in the contribution of discount types to the gross-to-net bubble is crucial for informing policymaking, as recent legislative proposals have aimed to eliminate commercial discounts from the insulin market, arguing that they distort market incentives and increase patient costs. However, the Congressional Budget Office determined that such an approach would increase insulin costs over time by discouraging net price competition.^4^ The decomposition of current discounts is therefore necessary to inform policymakers’ approach to addressing insulin affordability and to elucidate the implications of such policies for patients as well as the Medicaid and 340B programs.

We applied a newly published algorithm to decompose the overall gross-to-net bubble of leading insulin products into discount types.^5^ To our knowledge, this is the first study to decompose the overall gross-to-net bubble of these products. The algorithm accounted for the association between commercial discounts and mandatory discounts under the best-price provision, which requires drug manufacturers to provide the greatest available commercial discount to the Medicaid and 340B programs.^5,6^ The algorithm also considered the Medicaid rebate cap, which limits total Medicaid and 340B discounts to the mean price of the drug. Moreover, the algorithm isolated the coverage gap discounts under the Medicare Part D program. By properly attributing the overall discount to each category, we ascertained the relative magnitude of both commercial and mandatory discounts and clarified the association between growing commercial discounts and mandatory discounts.

## Methods

We selected the 4 predominant insulin products by Medicare Part D use: Lantus, Levemir, Humalog, and Novolog. We extracted the 2012 to 2019 data from 4 sources: (1) net sales and total units from SSR Health,^7^ (2) Medicare Part B and Part D 5% claims data, (3) Medicare Part D Prescriber Public Use File, and (4) Medicare and Medicaid Drug spending dashboards.^8,9^ The University of California, San Diego Institutional Review Board deemed this study exempt from review and waived the informed consent requirement because only deidentified or publicly available data were used.

### Estimation of the Gross-to-Net Bubble

Gross-to-net bubble was estimated by calculating the difference between the gross and net sales and represents total discounts for a given insulin product. Gross sales were estimated by multiplying the list price by the total number of units sold each year across the US, data that were obtained from Symphony Health.^10^ Net sales were obtained from SSR Health and represent the company-reported net revenue per product.^7^ Because drug manufacturers report net sales at the product family level, these estimates were inclusive of all formulations and doses within each product family. To account for the heterogeneity of formulations within a product, the list price was annually weighted by the relative use of each product formulation in Medicare Part D. In the estimation of 2019 data for Humalog, we subtracted sales for the authorized generic of insulin lispro from sales for branded Humalog. This estimation was necessary because Eli Lilly’s 2019 company reports bundled sales for the branded and authorized generic product in the reporting of net sales data.^11^

### Decomposition of the Gross-to-Net Bubble Into Discount Types

We decomposed the gross-to-net bubble (total discounts) into 4 discount types: (1) coverage gap discounts in Medicare Part D; (2) Medicaid discounts; (3) 340B discounts; and (4) commercial discounts, manufacturer discounts that were negotiated in commercial and Medicare Part D markets. Given data limitations, discounts to the US Department of Defense, Department of Veterans Affairs, or other federal programs were captured under commercial discounts. From 2014 to 2021, these discount programs accounted for 3.5% of total national health expenditures on retail prescription drugs and, for 1 manufacturer, represented 4% of total discounts provided^12,13^; therefore, we expected the consequences of this approach to be minimal for point estimates and to be consistent across time, limiting changes in patterns.

We used Medicare Part D claims data from a 5% random sample of Medicare beneficiaries and extracted all prescriptions for each insulin product filled every year. We estimated the total discount provided by the manufacturer under the Medicare Coverage Gap Discount Program using the reported gap discount amount and extrapolated the results to the entire Medicare population.

Medicaid discounts were estimated as the number of units reimbursed by Medicaid for a given insulin product (obtained from the Medicaid spending dashboard) multiplied by the Medicaid discount per unit. To estimate the Medicaid discount per unit, we followed a published method that accounts for best prices set by commercial discounts and the Medicaid rebate cap (eMethods in Supplement 1).^5^ In brief, we estimated the inflation penalty every year as the difference between the list price and the inflation-adjusted launch price.^5^ Then, we leveraged the relationship between commercial discounts and the Medicaid base rebate established by the best-price provision and estimated both simultaneously (eMethods in Supplement 1). This first iteration of the algorithm^5^ assumed that the commercial discount set best price. For observations and years when the commercial discount was less than 23.1% of the list price (rendering this assumption false), we used a second iteration of the algorithm^5^ to estimate the Medicaid base rebate as 23.1% of list price. Because Medicaid rebates are capped at 100% of the average manufacturer price, we tested for every year and insulin product whether the base discount and the inflation penalty exceeded the list price. For drug-year observations where the list price was exceeded, we applied a third iteration of the algorithm^5^ to estimate commercial discounts and Medicaid discounts when the Medicaid rebate cap applies (eMethods in Supplement 1).

The 340B discounts were estimated as the number of 340B eligible units multiplied by the 340B discount per unit. The 340B discount per unit equaled the Medicaid discount per unit and was estimated as described earlier. We estimated the number of 340B eligible units by extrapolating the proportion of Medicare units that were likely subject to 340B discounts to the overall market. To estimate the proportion of Medicare units that were likely subject to 340B discounts, we linked Medicare claims data to 340B-eligible prescribers and pharmacies, determining whether the prescription was written by a 340B-eligible prescriber and was filled at a 340B-eligible pharmacy. This analysis leveraged Medicare Parts B and D claims data, the Medicare Provider Utilization and Payment Data,^14^ and the lists of 340B-covered entities and contract pharmacies obtained from the Health Resources and Services Administration^15^ (eMethods in Supplement 1).

### Statistical Analysis

For each year and insulin product from 2012 to 2019, we reported the gross sales, net sales, gross-to-net bubble, commercial discounts, Medicare coverage gap discounts, Medicaid discounts, and 340B discounts in nominal US dollars. We identified years in which the estimated Medicaid and 340B discounts triggered the Medicaid rebate cap. We reported the proportion of gross sales accounted for by the gross-to-net bubble every year to describe temporal changes in the proportion of gross sales that was represented by discounts. We also reported the proportion of gross sales and gross-to-net bubble accounted for by each discount type over time. All results are shown at the product level instead of the national drug code level because manufacturers only report net sales data at the brand name level, which prevents the differentiation of net prices between formulations of a given insulin product. Analyses were conducted using SAS, version 9.4 (SAS Institute Inc) from June to December 2022.

## Results

### Changes in Gross Sales, Net Sales, and the Gross-to-Net Bubble

**Figure 1.**
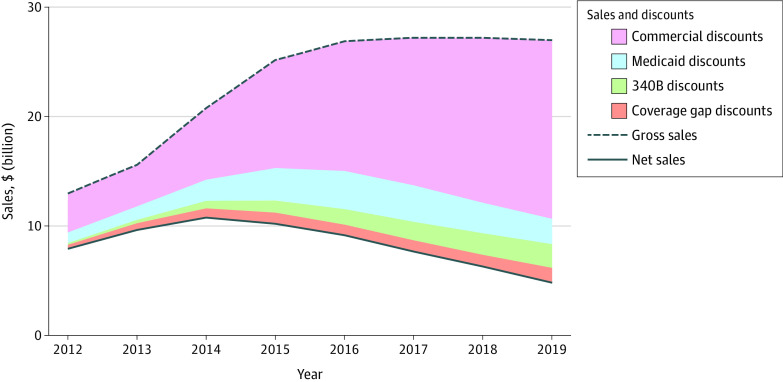
Decomposition of the Gross-to-Net Bubble Into Discount Types for 4 Insulin Products Combined Gross sales represent annual sales at list price before discounts are applied; net sales, annual revenue after discounts are applied; and gross-to-net bubble, sum of all discount types. Commercial discounts represent voluntary discounts that were negotiated between manufacturers and pharmacy benefit managers in the commercial and Medicare Part D markets. Numerical estimates are provided in eTable in Supplement 1.

Gross sales of the top 4 insulin products combined increased from $13.0 billion in 2012 to $27.0 billion in 2019 (Figure 1). Total discounts increased from nearly $4.9 billion to $22.0 billion, or 37.6% to 81.4% of gross sales. Subsequently, net sales decreased over the period, from $8.1 billion to $5.0 billion.

**Figure 2.**
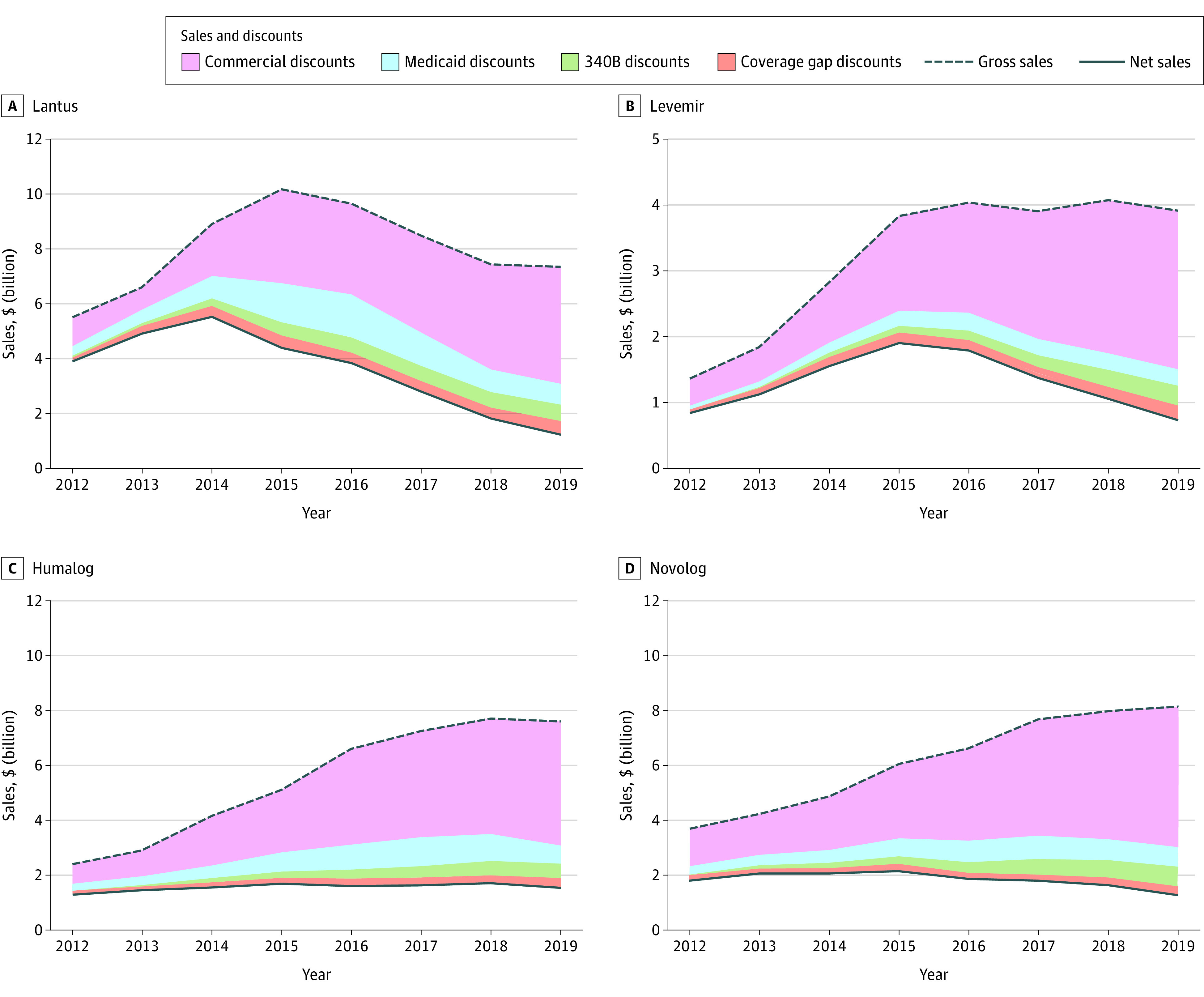
Decomposition of the Gross-to-Net Bubble Into Discount Types by Insulin Product Gross sales represent annual sales at list price before discounts are applied; net sales, annual revenue after discounts are applied; and gross-to-net bubble, sum of all discount types. Commercial discounts represent voluntary discounts that were negotiated between manufacturers and pharmacy benefit managers in the commercial and Medicare Part D markets. Numerical estimates are provided in eTable in Supplement 1.

Lantus gross sales increased from 2012 to 2015 and then decreased (Figure 2A; eTable in Supplement 1). Total discounts for Lantus quadrupled from $1.5 billion or 27.9% of gross sales in 2012 to $6.1 billion or 82.4% of gross sales in 2019 (Table 1). As a result, net sales for Lantus decreased by more than $4 billion after 2014.

**Table 1.** Proportion of Gross Sales Accounted for by Each Discount Type

Year	Gross sales, $, million ^a^	Gross-to-net bubble, $, million^b^	Gross-to-net bubble as % of gross sales, %	Discount amount^c^			
				Mandatory discounts, $, million (%)			Commercial discounts, $, million (%)^d^
				Coverage gap	340B	Medicaid	
**Four insulins combined**							
2012	12 974	4 880	37.6	261 (2.0)	160 (1.2)	962 (7.4)	3 497 (27.0)
2019	27 035	22 031	81.4	1 177 (4.4)	2 159 (8.0)	2 331 (8.6)	16 364 (60.5)
**Lantus**							
2012	5 499	1 532	27.9	119 (2.2)	54 (1.0)	347 (6.3)	1 012 (18.4)
2019	7 348	6 051	82.4	425 (5.8)	601 (8.2)	752 (10.2)	4 273 (58.2)
**Levemir**							
2012	1 360	492	36.2	30 (2.2)	9 (0.6)	58 (4.3)	396 (29.1)
2019	3 916	3 153	80.5	196 (5.0)	297 (7.6)	254 (6.5)	2 405 (61.4)
**Humalog**							
2012	2 429	1 058	43.6	49 (2.0)	40 (1.6)	246 (10.1)	724 (29.8)
2019	7 616	6 001	78.8	282 (3.7)	541 (7.1)	634 (8.3)	4 543 (59.7)
**Novolog**							
2012	3 687	1 798	48.8	63 (1.7)	58 (1.6)	311 (8.4)	1 366 (37.0)
2019	8 155	6 825	83.7	273 (3.3)	720 (8.8)	690 (8.5)	5 143 (63.1)

^a^ Gross sales represent annual sales for a product at list price before discounts are applied.

^b^ Gross-to-net bubble is the difference between gross and net sales and represents the sum of all discount types.

^c^ Discount amount is the proportion of gross sales accounted for by each discount type.

^d^ Commercial discounts represent voluntary discounts that were negotiated between manufacturers and pharmacy benefit managers in the commercial and Medicare Part D markets. Discounts to the US Department of Defense, Department of Veterans Affairs, or other federal programs are included under commercial discounts.

Levemir gross sales increased from 2012 to 2016 and then remained constant (Figure 2B). Total discounts for Levemir increased from $492 million or 36.2% of gross sales in 2012 to nearly $3.2 billion or 80.5% of gross sales in 2019 (Table 1). As a result, Levemir net sales decreased after 2015.

Humalog gross sales tripled from 2012 to 2018 (Figure 2C). Concurrently, discounts increased from $1.0 billion in 2012 or 43.6% of gross sales to $6.0 billion or 78.8% of gross sales in 2019 (Table 1). Novolog gross sales and discounts followed the pattern observed for Humalog (Figure 2D).

### Commercial and Mandatory Discounts as a Proportion of Gross Sales

As a proportion of gross sales, commercial discounts more than doubled, increasing from 27.0% of gross sales in 2012 or $3.5 billion to 60.5% or $16.4 billion in 2019 (Table 1). Mandatory discounts represented 10.7% of gross sales in 2012 (or $1.4 billion) compared with $5.7 billion or 21.0% of gross sales in 2019. Coverage gap discounts increased from 2.0% of gross sales to 4.4% over the period, whereas Medicaid rebates increased from 7.4% to 8.6% of gross sales. The 340B discounts increased from 1.2% to 8.0% of gross sales from 2012 to 2019. During this period, all 4 insulin products triggered the Medicaid rebate cap (Novolog in 2013, Humalog in 2014, Lantus in 2015, and Levemir in 2019), and Medicaid use increased from 9.4% of units sold across the 4 insulin products in 2012 to 13.4% of units in 2016 before decreasing to 8.7% of units in 2019. The 340B use increased from 1.5% of units sold in 2012 to 7.4% of units sold in 2019.

The 4 drugs were similar in the distribution of discount types. Therefore, we limited the in-text reporting of specific results to Lantus, as it was the insulin with the highest gross sales over the study period.

Commercial discounts increased substantially in both dollar value and proportion of gross sales represented (Table 1). For instance, for Lantus, commercial discounts increased from 18.4% in 2012 or $1.0 billion to 58.2% or $4.2 billion in 2019.

Among mandatory discounts, 340B discounts increased the most over the study period. For Lantus, 340B discounts increased from 1.0% of gross sales in 2012 to 8.2% in 2019, consistent with growth in use (1.5% of units sold in 2012 to 8.2% in 2019). Medicaid discounts varied over the period; for Lantus, Medicaid discounts increased from 6.3% of gross sales in 2012 to 16.0% in 2016, when they peaked, decreasing to 10.2% in 2019. Medicaid discounts decreased after the Medicaid rebate cap was triggered in 2015 and use was reduced (Medicaid represented 16.1% of units sold in 2016 vs 10.2% in 2019). For Lantus, coverage gap discounts increased from 2.2% of gross sales in 2012 to 5.8% in 2019.

### Commercial and Mandatory Discounts as a Proportion of Total Discounts

**Figure 3.**
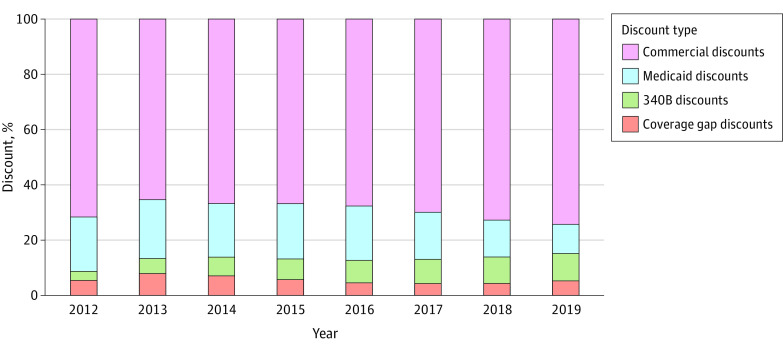
Proportion of the Gross-to-Net Bubble Accounted for by Each Discount Type for 4 Insulin Products Combined Commercial discounts represent voluntary discounts that were negotiated between manufacturers and pharmacy benefit managers in the commercial and Medicare Part D markets.

From 2012 to 2019, most of the discounts were commercial discounts, increasing from 71.7% of the gross-to-net bubble to 74.3% (Figure 3). Mandatory discounts represented the remaining 26.7% of total discounts in 2019 vs 29.3% in 2012. Among mandatory discounts, coverage gap discounts remained relatively consistent as a proportion of all discounts over the period, accounting for 5.4% of the gross-to-net bubble in 2012 and 5.3% in 2019. Medicaid discounts decreased as a proportion of total discounts over the period from 19.7% to 10.6%. The 340B discounts increased as a proportion of total discounts from 3.3% in 2012 to 9.8% in 2019.

Results for each insulin product were similar and consistent with the results for the 4 insulin products combined (Table 2). For instance, for Lantus, commercial discounts increased from 66.1% of total discounts in 2012 to 70.6% in 2019, the 340B discounts increased from 3.5% to 9.9%, and Medicaid discounts decreased from 22.6% to 12.4%. Coverage gap discounts were consistent at 7.8% in 2012 and 7.0% in 2019.

**Table 2.** Proportion of the Gross-to-Net Bubble Accounted for by Each Discount Type by Insulin Product

	Proportion of the gross-to-net bubble accounted for by each discount type, %			
	Mandatory discounts			Commercial discounts^a^
	Coverage gap	340B	Medicaid	
**Lantus**				
2012	7.8	3.5	22.6	66.1
2019	7.0	9.9	12.4	70.6
**Levemir**				
2012	6.1	1.8	11.8	80.4
2019	6.2	9.4	8.1	76.3
**Humalog**				
2012	4.6	3.7	23.2	68.4
2019	4.7	9.0	10.6	75.7
**Novolog**				
2012	3.5	3.2	17.3	76.0
2019	4.0	10.5	10.1	75.3

^a^ Commercial discounts represent voluntary discounts that were negotiated between manufacturers and pharmacy benefit managers in the commercial and Medicare Part D markets. Discounts to the US Department of Defense, Department of Veterans Affairs, or other federal programs are included under commercial discounts.

## Discussion

In this decomposition of the gross-to-net bubble for leading insulin products, we characterized the growing role of commercial discounts in lowering net sales compared with mandatory discounts under the Medicare coverage gap, Medicaid, and 340B programs. While both commercial and mandatory discounts increased over the study period as a proportion of gross sales, commercial discounts increased at a faster rate than mandatory discounts. These patterns demonstrated that commercial discounts are a greater contributing factor in reduced net sales than mandatory discounts. Mandatory discounts declined as a proportion of total discounts over the period, rebutting manufacturer arguments that mandatory discounts play a role in rising gross-to-net bubble for insulin products.

These divergent growth rates may be explained, in part, by the cap on mandatory discounts in the Medicaid and 340B programs. While the Medicaid and 340B discounts depend on commercial discounts under the best-price requirement, all 4 insulin products triggered the rebate cap over the period, allowing manufacturers to take greater list price increases and offer more commercial discounts without affecting net sales to mandatory programs. Across the 4 insulin products, Medicaid units as a proportion of total units changed little from 2012 to 2019 (9.4% to 8.7%), but Medicaid rebates as a proportion of all discounts decreased by nearly half (19.7% to 10.6%) even as the commercial discounts and inflation penalties used to calculate the Medicaid rebate increased. This association underscored the role of the Medicaid rebate cap in limiting the role of mandatory discounts in decreasing net sales for drug products. This cap also limits the role of the Medicaid inflation penalty in discouraging drug price increases above the rate of inflation^16^; recognizing this association, lawmakers eliminated the cap beginning in 2024.^17^

In the past few months, Sanofi, Eli Lilly, and Novo Nordisk announced reductions in the list prices of their legacy insulin products.^18,19,20^ Specifically, Sanofi announced a 78% price reduction for Lantus; Novo Nordisk, a 65% price reduction for Levemir and 75% reduction for Novolog; and Eli Lilly, a 70% price reduction for Humalog.^18,19,20^These price reductions have been partially attributed to the lifting of the Medicaid rebate cap in 2024, under which manufacturers would have had to pay rebate penalties to Medicaid.^21^ By decreasing the list price and presumably rebates, manufacturers will lower base rebates under the best-price provision and inflation rebates that are triggered by year-over-year increases in list prices above inflation. The announced price reductions are greater than the estimated commercial discounts for 2019, suggesting that commercial price competition has continued to reduce net prices since 2019.

Despite the announced price reductions for the top insulin products, the findings of this analysis remain relevant to policymakers as they contextualize the extent to which the gross-to-net bubble represents the commercial discounts offered by manufacturers vs the mandatory discounts that manufacturers frequently allege as the reason for lower net sales. The decomposition of mandatory and commercial discounts found that the widening gap between list and net prices was primarily associated with pharmaceutical benefit managers’ negotiation of commercial discounts in exchange for formulary placement. These increasing discounts reduced total spending on insulin, although they likely exacerbated inequities in access because commercial discounts did not lower insulin list prices for patients without insurance.

We did not measure patterns in beneficiary cost-sharing as such analyses were outside of the scope of this quantification of manufacturer discounts. Patterns in cost-sharing for insulin products among Medicare Part D beneficiaries can, however, be ascertained from data reported by the Centers for Medicare & Medicaid Services.^22^ These data showed that Medicare beneficiary cost-sharing for insulin products decreased in relative terms (as a proportion of total claim costs) but increased in absolute value.^22^ For example, Medicare Part D beneficiary cost-sharing decreased from 10.5% of total claim costs in 2013 to 7.5% in 2019 for Lantus and from 12.5% to 5.7% for Levemir.^22^ However, nominal mean beneficiary cost-sharing increased by 19.6% for Lantus and by 23.1% for Levemir, which may be partially attributable to changes in benefit design, as the standard Medicare Part D deductible increased by 27.7% over this period.^23,24^

### Limitations

This study has 4 main limitations. First, the analysis was limited to insulin products, whose prices have been the target of intense scrutiny. Future research should examine the patterns in commercial and mandatory discounts in other costly drug classes. Second, the methods underpinning the analysis did not separately distinguish discounts or sales units to the Department of Defense, Department of Veterans Affairs, or other federal programs, which were therefore included under commercial discounts. This inclusion may lead to an overestimation of commercial discounts. However, given the relatively small share of sales attributable to these programs, we believe the impact was minimal.

Third, the analysis used the mean commercial discount to estimate best price for Medicaid and 340B program discounts, but in practice, the greatest commercial discount establishes best price. We may, therefore, understate Medicaid and 340B discounts in years when the discount does not trigger the Medicaid rebate cap. However, because the rebate cap was triggered for most years in the sample for most drugs, the impact of this limitation was likely minimal. Fourth, due to the unavailability of Medicaid and commercial insurance claims, we used Medicare claims to estimate the proportion of units that were eligible for 340B discounts, as was previously done.^5^ We then extrapolated this proportion of units eligible for 340B discounts to the Medicaid and commercial insurance markets. While this algorithm^5^ may have introduced estimation errors, it was superior to the bundling of 340B discounts with commercial discounts in prior estimates of net prices. Regardless of these limitations, the algorithm^5^ is a substantial contribution to the study of pharmaceutical pricing and reimbursement, as it estimates for the first time the relative contribution of pharmaceutical discount types in the gross-to-net bubble. This method provides policymakers with more precise knowledge of opaque discounting practices, which is necessary for the development of policies to address drug affordability without increasing overall spending.

## Conclusion

In this analysis of the gross-to-net bubble, from 2012 to 2019, discounts on leading insulin products increased from 37.6% to 81.4% of gross sales. The majority of the discounts were commercial discounts that were negotiated with insurers and pharmaceutical benefit managers, which increased from 27.0% of gross sales to 60.5% of gross sales over the study period. Mandatory discounts under federal programs, including Medicare Part D coverage gap, Medicaid, and 340B, remained less than 30% of all discounts over the period, with Medicaid rebates accounting for most of the discounts. As policymakers consider options to make insulin more affordable, they should consider the growing role of commercial discounts provided by drug manufacturers in reducing net sales compared with mandatory discounts required under federal programs.

## Notes

Supplement 1.
eMethods
**eTable.** Decomposition of the Gross-to-Net Bubble Into Discount Types, by Insulin Product
eReferences

Supplement 2.
Data Sharing Statement
