# Reference Omitted

**DOI:** 10.1001/jamanetworkopen.2023.26531

**Published:** 2023-07-17

In the Original Investigation titled “Assessment of Commercial and Mandatory Discounts in the Gross-to-Net Bubble for the Top Insulin Products From 2012 to 2019”^1^ published online June 14, 2023, a reference was omitted from the reference list. This article has been corrected.
