# Supplementary material for: Assessment of Commercial and Mandatory Discounts in the Gross-to-Net Bubble for the Top Insulin Products From 2012 to 2019
Source: JAMA Netw Open. 2023 Jun 14;6(6):e2318145. doi: 10.1001/jamanetworkopen.2023.18145 (PMC10267767; doi:10.1001/jamanetworkopen.2023.18145)
Supplement: Supplement 1. — eMethods eTable. Decomposition of the Gross-to-Net Bubble Into Discount Types, by Insulin Product eReferences [file jamanetwopen-e2318145-s001.pdf]

Supplemental Online Content

Dickson SR, Gabriel N, Gellad WF, Hernandez I. Assessment of commercial and mandatory discounts in the gross-to-net bubble for the top insulin products from 2012 to 2019. *JAMA Netw Open*. 2023;6(6):e2318145. doi:10.1001/jamanetworkopen.2023.18145

eMethods

eTable. Decomposition of the Gross-to-Net Bubble Into Discount Types, by Insulin Product

eReferences

This supplemental material has been provided by the authors to give readers additional information about their work.

© 2023 Dickson SR et al. *JAMA Network Open*

eMethods

Estimation of Commercial Discounts and Medicaid Discounts

Our methodology relies on the relationship between commercial discounts and Medicaid discounts established by the Best price provision, as previously used in the peer-reviewed literature.[1]

First, we estimate Medicaid discounts as follows:

Medicaid discounts= number of Medicaid units * Medicaid discount per unit

Number of Medicaid units were obtained from the Centers for Medicare and Medicaid Services dashboard.

Medicaid discount per unit=base rebate + inflation penalty

- The inflation penalty was calculated for each product and year as the difference between the list price and the inflation-adjusted launch price, following a published method.[2] Launch price data is available at the national drug code level and our analyses were conducted at the product level. To estimate an inflation-adjusted launch price at the product level, we calculated the inflation-adjusted launch price for each national drug code every year, and weighted by the relative utilization of each national drug code every year.
- The base rebate is calculated as the greater of 23% discount or the Best Price offered to any purchaser. To estimate the base rebate, in the first iteration of our algorithm, we assumed that the commercial discount set Best Price. Therefore, we subtracted the inflation Medicaid and 340B inflation penalty discounts from the gross-to-net-bubble.

  Gross-to-net-bubble- Medicaid inflation penalty- 340B inflation penalty-coverage gap discounts=**x**

  We amortize the remaining discount **x** amount across the sum of group health plans, Part D, Medicaid, and 340B program units to generate the estimated commercial discount per unit. Then, the total commercial discount is estimated as the product of the commercial discount per unit and commercial units (group health insurance and Medicare Part D units).

  If the estimated commercial discount ≥23.1% of list price, then the assumption that commercial discount set Best Price holds.

  If the estimated commercial discount < 23.1% of list price, then the commercial discount does not trigger the Best Price provision. In this case, we re-estimate Medicaid and 340B discounts as the sum of 23.1% of list price and inflation penalty. We subtract these re-calculated discounts to the Medicaid program and 340B discounts from the gross-to-net bubble. The resulting estimate **y** represents commercial discounts.

  Gross-to-net-bubble- Medicaid units *(23% of list price + inflation penalty)- 340B units* (23% of list price + inflation penalty)-coverage gap discounts=**y**

Medicaid and 340B discounts are capped at 100% of the Average Manufacturer Price, in other words, the sum of the base discount and the inflation penalty cannot exceed the invoice price. We checked whether the sum of the estimated base rebate and inflation penalty exceeded the list price. For drug-year observations where it did, we recalculated the total amount of commercial discounts as follows, where **z** represents commercial discounts:

Gross-to-net-bubble- Medicaid units*list price-340B units * list price-coverage gap discounts=**z**

© 2023 Dickson S et al. *JAMA Network Open*

Estimation of 340B Discounts

340B discounts= number of units subject to 340B discounts * 340B discount per unit

340B discount per unit= Medicaid discount per unit (estimated as described above)

Number of units subject to 340B discounts= Medicare Part B 340B units + commercial 340B units (where commercial includes group health insurance plans and Medicare Part D)

- Medicare Part B 340B Units = Medicare Part B Units * Proportion of Medicare Part B units subject to 340B discounts
    - Medicare Part B Units were obtained from the Centers for Medicare and Medicaid Services spending dashboard ‡
    - To estimate the proportion of Medicare Part B units subject to 340B discounts, we used Part B claims from a 5% random sample of Medicare beneficiaries and a previously published approach.[2] In brief, we extracted all Part B claims for the sample of drugs and identified those originating from 340B institutions by matching claims to the list of 340B institutions from the Health Resources & Services Administration (HRSA).[3] Carrier claims were matched using the national provider identifier and the address and outpatient claims using the Medicare Provider number.
- Commercial 340B units = commercial units prescribed at 340B eligible entities* proportion of units prescribed at 340B eligible entities that are filled at 340B pharmacies.
    - Commercial units prescribed at 340B eligible entities were estimated by matching the Medicare Part D Prescriber Utilization File[4] to the 340B Covered Entity File for each year.[2]
    - To determine the proportion of 340B-Prescribed Units that were filled at 340B pharmacies, we first extracted pharmacy claims from a 5% random sample of Medicare beneficiaries that were prescribed by prescribers identified as 340B-eligible, using the methodology described above. Then, we calculated the proportion of these claims that were dispensed at 340B pharmacies by matching the dispensing pharmacy in each claim to the 340B Pharmacy File obtained from Health Resources & Services Administration.[3]

© 2023 Dickson S et al. *JAMA Network Open*

eTable. Decomposition of the Gross-to-net Bubble into Discount Types, by Insulin Product

Lantus	Gross Sales [a]	Net Sales	Gross-to-Net Bubble [b]	Coverage Gap Discounts	340B Discounts	Medicaid Discounts	Commercial Discounts [c]
2012	$5499 M	$3967 M	$1532 M	$119 M	$54 M	$347 M	$1012 M
2013	$6596 M	$4977 M	$1619 M	$212 M	$113 M	$479 M	$814 M
2014	$8902 M	$5597 M	$3306 M	$326 M	$270 M	$829 M	$1881 M
2015	$10168 M	$4465 M	$5703 M	$381 M	$490 M	$1414 M	$3417 M
2016	$9649 M	$3902 M	$5746 M	$319 M	$549 M	$1554 M	$3324 M
2017	$8467 M	$2864 M	$5604 M	$318 M	$559 M	$1198 M	$3528 M
2018	$7431 M	$1908 M	$5524 M	$309 M	$563 M	$822 M	$3830 M
2019	$7348 M	$1296 M	$6051 M	$425 M	$601 M	$752 M	$4273 M
**Levemir**							
2012	$1360 M	$868 M	$492 M	$30 M	$9 M	$58 M	$396 M
2013	$1849 M	$1155 M	$694 M	$62 M	$23 M	$78 M	$531 M
2014	$2827 M	$1586 M	$1241 M	$105 M	$65 M	$149 M	$922 M
2015	$3842 M	$1930 M	$1913 M	$141 M	$103 M	$218 M	$1450 M
2016	$4044 M	$1820 M	$2223 M	$133 M	$141 M	$265 M	$1685 M
2017	$3915 M	$1410 M	$2505 M	$134 M	$178 M	$245 M	$1948 M
2018	$4083 M	$1091 M	$2992 M	$153 M	$259 M	$246 M	$2334 M
2019	$3916 M	$763 M	$3153 M	$196 M	$297 M	$254 M	$2405 M
**Humalog**							
2012	$2429 M	$1371 M	$1058 M	$49 M	$40 M	$246 M	$724 M
2013	$2919 M	$1521 M	$1398 M	$82 M	$68 M	$303 M	$945 M
2014	$4180 M	$1628 M	$2552 M	$123 M	$153 M	$460 M	$1816 M
2015	$5134 M	$1772 M	$3362 M	$149 M	$223 M	$696 M	$2294 M
2016	$6624 M	$1685 M	$4939 M	$191 M	$343 M	$897 M	$3508 M
2017	$7268 M	$1718 M	$5550 M	$211 M	$420 M	$1034 M	$3886 M
2018	$7716 M	$1788 M	$5928 M	$218 M	$541 M	$948 M	$4221 M
2019	$7616 M	$1615 M	$6001 M	$282 M	$541 M	$634 M	$4543 M
**Novolog**							
2012	$3687 M	$1889 M	$1798 M	$63 M	$58 M	$311 M	$1366 M
2013	$4250 M	$2139 M	$2111 M	$113 M	$105 M	$380 M	$1513 M
2014	$4886 M	$2141 M	$2746 M	$143 M	$180 M	$461 M	$1962 M
2015	$6081 M	$2224 M	$3857 M	$177 M	$296 M	$645 M	$2739 M
2016	$6643 M	$1943 M	$4699 M	$146 M	$390 M	$779 M	$3384 M
2017	$7710 M	$1862 M	$5847 M	$184 M	$545 M	$865 M	$4253 M
2018	$8022 M	$1700 M	$6322 M	$206 M	$629 M	$774 M	$4713 M
2019	$8155 M	$1330 M	$6825 M	$273 M	$720 M	$690 M	$5143 M

© 2023 Dickson S et al. *JAMA Network Open*

eTable cont.

	Gross Sales [a]	Net Sales	Gross-to-Net Bubble[b]	Coverage Gap Discounts	340B Discounts	Medicaid Discounts	Commercial Discounts [c]
**All Four Insulins Combined**							
2012	$12974 M	$8094 M	$4880 M	$261 M	$160 M	$962 M	$3497 M
2013	$15614 M	$9793 M	$5822 M	$468 M	$310 M	$1240 M	$3803 M
2014	$20795 M	$10951 M	$9844 M	$697 M	$668 M	$1898 M	$6581 M
2015	$25226 M	$10391 M	$14835 M	$848 M	$1112 M	$2974 M	$9901 M
2016	$26959 M	$9351 M	$17608 M	$790 M	$1423 M	$3495 M	$11900 M
2017	$27360 M	$7854 M	$19506 M	$847 M	$1702 M	$3343 M	$13614 M
2018	$27252 M	$6487 M	$20765 M	$886 M	$1992 M	$2789 M	$15098 M
2019	$27035 M	$5004 M	$22031 M	$1177 M	$2159 M	$2331 M	$16364 M

[a] Gross sales represent annual sales for a product at list price, before discounts are applied.

[b] The gross-to-net-bubble is the difference between gross and net sales and represents the sum of all discount types.

[c] Commercial discounts represent voluntary discounts negotiated between manufacturers and Pharmacy Benefit Managers in the commercial and Medicare Part D markets. Discounts to the Department of Defense, Department of Veterans Affairs, or other Federal programs are included under commercial discounts, as acknowledged in the limitations.

© 2023 Dickson S et al. *JAMA Network Open*

eReferences

1. Dickson S, Gabriel N, Hernandez I. Estimated Changes in Price Discounts for Tenofovir-Inclusive HIV Treatments Following Introduction of Tenofovir Alafenamide. *AIDS 2022 doi: 101097/QAD0000000000003401*.

2. Dickson S, Gabriel N, Gellad W, Hernandez I. Reduction in Medicaid rebates paid by pharmaceutical manufacturers for outpatient injected, inhaled, infused, implanted, or instilled drugs: The 5i loophole. *J Health Polit Policy Law* 2022. doi:10.1215/03616878-10041219

3. Health Resources & Services Administration. 340B Drug Pricing Program. Accessed September 14, 2022. https://www.hrsa.gov/opa

4. Medicare Provider Utilization and Payment Data: Part D Prescriber. Accessed September 14, 2022. https://data.cms.gov/provider-summary-by-type-of-service/medicare-part-d-prescribers

© 2023 Dickson S et al. *JAMA Network Open*
