# Supplementary material for: Assessment of Commercial and Mandatory Discounts in the Gross-to-Net Bubble for the Top Insulin Products From 2012 to 2019
Source: JAMA Netw Open. 2023 Jun 14;6(6):e2318145. doi: 10.1001/jamanetworkopen.2023.18145 (PMC10267767; doi:10.1001/jamanetworkopen.2023.18145)
Supplement: Supplement 2. — Data Sharing Statement [file jamanetwopen-e2318145-s002.pdf]

Data Sharing Statement

Dickson. Assessment of Commercial and Mandatory Discounts in the Gross-to-Net Bubble for the Top Insulin Products From 2012 to 2019. *JAMA Netw Open*. Published June 14, 2023. doi:10.1001/jamanetworkopen.2023.18145

Data
Data available: No

Additional Information
Explanation for why data not available: Data were obtained under a data user agreement that prohibits data sharing
